# Induction of neuropilin-1 and vascular endothelial growth factor by epidermal growth factor in human gastric cancer cells

**DOI:** 10.1038/sj.bjc.6600811

**Published:** 2003-03-04

**Authors:** M Akagi, M Kawaguchi, W Liu, M F McCarty, A Takeda, F Fan, O Stoeltzing, A A Parikh, Y D Jung, C D Bucana, P F Mansfield, D J Hicklin, L M Ellis

**Affiliations:** 1 Department of Cancer Biology, The University of Texas MD Anderson Cancer Center, 1515 Holcombe Boulevard, Houston, TX 77030, USA; 2 Department of Surgical Oncology, The University of Texas MD Anderson Cancer Center, 1515 Holcombe Boulevard, Houston, TX 77030, USA; 3 ImClone Systems, Inc., 180 Varick Street, New York, NY 10014, USA

**Keywords:** gastric cancer, angiogenesis, epidermal growth factor, neuropilin, vascular endothelial growth factor, signal transduction

## Abstract

The epidermal growth factor receptor (EGF-R) pathway plays a pivotal role in the progression of human gastric cancer. The angiogenic factor vascular endothelial growth factor (VEGF) has been shown to be induced by EGF in various cancer cell lines. Neuropilin-1 (NRP-1) acts as a coreceptor for VEGF-165 and increases its affinity for VEGF receptor 2 (VEGFR-2) in endothelial cells. Furthermore, NRP-1 has been found to be expressed by tumour cells and has been shown to enhance tumour angiogenesis and growth in preclinical models. We examined the expression of NRP-1 mRNA and EGF-R protein in seven human gastric cancer cell lines. NRP-1 expression was expressed in five of seven cell lines, and EGF-R expression closely mirrored NRP-1 expression. Moreover, in EGF-R-positive NCI-N87 and ST-2 cells, EGF induced both NRP-1 and VEGF mRNA expression. C225, a monoclonal antibody to EGF-R, blocked EGF-induced NRP-1 and VEGF expression in NCI-N87 cells in a dose-dependent manner. The treatment of NCI-N87 cells with EGF resulted in increases in phosphorylation of Erk1/2, Akt, and P38. Blockade of the Erk, phosphatidylinositol-3 kinase/Akt, or P38 pathways in this cell line prevented EGF induction of NRP-1 and VEGF. These results suggest that regulation of NRP-1 expression in human gastric cancer is intimately associated with the EGF/EGF-R system. Activation of EGF-R might contribute to gastric cancer angiogenesis by a mechanism that involves upregulation of VEGF and NRP-1 expression via multiple signalling pathways.

The growth of human gastric cancer cells involves a variety of growth factors, gut hormones, and cytokines (Tahara *et al*, 1993). In particular, the epidermal growth factor receptor (EGF-R) pathway appears to play a crucial role in gastric cancer progression. A large percentage of gastric cancer cell lines express EGF-R (Yokozaki, 2000), and gastric cancer cells grow in response to EGF/transforming growth factor-*α* (TGF-*α*) activation of EGF-R in an autocrine loop (Yoshida *et al*, 1990; Piontek *et al*, 1993). Expression of EGF and its receptor has been found to correlate with prognosis in patients with gastric cancer (Yasui *et al*, 1988; Jonjic *et al*, 1997).

Tumour angiogenesis is essential for the growth and metastasis of solid tumours, and the process of angiogenesis is mediated by numerous stimulatory and inhibitory factors (Folkman, 1995). One such factor is vascular endothelial growth factor (VEGF), a potent mitogenic and chemotactic factor for endothelial cells (ECs) *in vitro* and an angiogenic factor *in vivo* (Leung *et al*, 1989; Kondo *et al*, 2000). The expression of VEGF has been correlated with tumour progression and poor clinical outcome in various cancer systems including gastric cancer (Maeda *et al*, 1996; Takahashi *et al*, 1996; Kido *et al*, 2001). VEGF is expressed as four isoforms derived from alternate splicing of the mRNA (Tischer *et al*, 1991). The smaller isoforms, VEGF-121 and VEGF-165, are secreted with VEGF-165 being the predominant isoform in most tumours. The classic receptors for VEGF possess tyrosine kinase activity and are expressed primarily on ECs. The current nomenclature for the VEGF receptors lists three receptors: VEGFR-1 (flt-1), VEGFR-2 (kdr/flk-1), and VEGFR-3 (flt-4) (Fournier *et al*, 1997). VEGF-induced mitogenesis and angiogenesis are mediated largely by VEGFR-2 (Veikkola *et al*, 2000).

Neuropilin-1 (NRP-1) was first described as a semaphorin receptor important for the guidance of developing neurons (He and Tessier-Lavigne, 1997; Kolodkin *et al*, 1997). Transgenic overexpression or knockout of the *NRP-1* gene results in lethal abnormalities in the cardiovascular system, suggesting that NRP-1 plays a role in vasculogenesis and possibly angiogenesis (Kitsukawa *et al*, 1995; Kawasaki *et al*, 1999). More recently, NRP-1 has been found to be expressed on ECs, and coexpression of NRP-1 and VEGFR-2 on ECs enhances the biological activity of VEGFR-2 in response to the VEGF-165 isoform (Soker *et al*, 1998; Whitaker *et al*, 2001). These findings suggest that NRP-1 acts as a coreceptor for VEGFR-2 in ECs and functions in VEGF-mediated angiogenesis and vasculogenesis. NRP-1 is also expressed by several types of tumour cells, such as breast and prostate cancers (Soker *et al*, 1998), and overexpression of NRP-1 in prostate carcinoma cells has been shown to enhance tumour angiogenesis and growth (Miao *et al*, 2000).

Experimental evidence suggests a role for the EGF ligand–receptor system in the induction of angiogenesis. In fact, it has been shown that EGF and TGF-*α* can upregulate the production of VEGF, currently regarded as the major proangiogenic factor for most types of human cancer (Goldman *et al*, 1993; Kerbel *et al*, 1998). A recent study has shown that EGF induces NRP-1 mRNA expression in astrocytoma (Ding *et al*, 2000), but the expression and regulation of NRP-1 in gastric cancer has not previously been investigated. We hypothesized that EGF, as a regulator of other angiogenic factors such as VEGF in other tumour types, regulates both VEGF and NRP-1 in human gastric cancer cell lines. In this study, we examined NRP-1 and EGF-R expression in human gastric cancer cell lines and evaluated the effect of EGF on NRP-1 and VEGF expression. We also investigated major signalling pathways involved in NRP-1 and VEGF induction by EGF.

## MATERIALS AND METHODS

### Materials

Recombinant human EGF was purchased from R&D Systems, Inc. (Minneapolis, MN, USA). The anti-human EGF-R monoclonal antibody C225 was provided by ImClone Systems (New York, NY, USA). The mitogen-activated protein kinase (MAPK)/extracellular signal-regulated kinase (MEK) 1/2 inhibitor U0126 and MEK1 inhibitor PD98059 were obtained from New England Biolabs Inc. (Beverly, MA, USA). The phosphatidylinositol-3 (PI-3) kinase/Akt inhibitor wortmannin was purchased from Sigma Chemical Company (St Louis, MO, USA). The P38 MAPK inhibitor SB203580 was purchased from Calbiochem (San Diego, CA, USA). Diaminobenzidine substrate (DAB) and Universal Mount was purchased from Research Genetics (Huntsville, AL, USA). Superfrost slides were purchased from Fisher Scientific Co. (Houston, TX, USA). Streptavidin HRP was purchased from DAKO (Carpinteria, CA, USA), TSA Biotin System was purchased from Perkin Elmer Life Science Inc. (Boston, MA, USA). Antibodies for the immunohistochemical analysis were obtained as follows: rabbit anti-NRP-1 antibody from Santa Cruz (Santa Cruz, CA, USA); mouse anti-EGF-R monoclonal antibody from Zymed (South San Francisco, CA, USA); biotinated goat anti-mouse IgG from Biocare Medical (Walnut Creek, CA, USA); peroxidase-conjugated goat anti-rabbit IgG from Jackson Immuno-Research Laboratories (West Grove, PA, USA).

### Cell lines and culture conditions

The human gastric carcinoma cell lines AGS and NCI-N87 were obtained from American Type Culture Collection (Manassas, VA, USA). The ST-2, ST-4, and ST-8 human gastric cancer cells were established from patients at The University of Texas MD Anderson Cancer Center (a gift from Dr Bradley McIntyre) (Yadav *et al*, 1996). TMK-1 cells were a gift from Dr Eiichi Tahara (Hiroshima University School of Medicine, Hiroshima, Japan) (Ito *et al*, 1989). KKLS cells were kindly provided by Dr Yutaka Takahashi (Cancer Research Institute, Kanazawa University, Kanazawa, Japan).

The cell lines were grown in minimum essential medium or RPMI 1640 (Life Technologies, Grand Island, NY, USA) supplemented with 10% foetal bovine serum (FBS) and 2 U ml^−1^ penicillin – streptomycin. All experiments were performed at subconfluence (70–80%) to avoid variations in VEGF expression due to confluence (Koura, 1996). Before treatment, cells were incubated in 5% FBS-containing medium overnight to minimise the effect of serum starvation on VEGF induction (Jung *et al*, 1999).

### Reverse transcriptase–polymerase chain reaction

Total RNA was extracted from human gastric carcinoma cell lines using Tri Reagent (Molecular Research Center, Inc., Cincinnati, OH, USA) following the manufacturer's instructions. For reverse transcriptase–polymerase chain reaction (RT–PCR), 3 *μ*g of total RNA was used for cDNA synthesis with avian myeloblastosis virus reverse transcriptase (Life Technologies) in a final volume of 20 *μ*l. The reaction mixture included 0.5 M Tris-HCl (pH 8.0), 0.5 M KCl, 0.05 M MgCl_2_, 2.5 mM dNTP, 40 U of RNase inhibitor (Boehringer Mannheim, Indianapolis, IN, USA), 50 U of reverse transcriptase, and 0.5 *μ*g of random primers. The cDNA synthesis reaction was performed for 1 h at 42°C. A portion of the reaction mixture (5 *μ*l) was subjected to PCR amplification in a reaction mixture (50 *μ*l) that contained 1 *μ*mol l^−1^ of each of two primers (sense and antisense), 1.5 mmol l^−1^ of MgCl_2_, 0.2 mmol l^−1^ of each of four deoxynucleotides, and 2.5 U of *Taq* polymerase (Promega, Madison, WI, USA). PCR amplification was performed under the following conditions: 94°C for 5 min; 35 cycles of 1 min denaturing at 94°C, 30 s of annealing at 57°C, and 1 min of extension at 72°C. PCR products were analysed by electrophoresis of 20 *μ*l of each PCR reaction mixture in a 3% agarose gel, and bands were visualised by ethidium bromide staining. The primers chosen were as follows: NRP-1 sense, 3′-ACGATGAATGTGGCGATACT-5′; antisense, 5′-AGTGCATTCAAGGCTGTTGG-3′. Human umbilical vein endothelial cell RNA was used as a positive control.

### Determination of EGF's effects on NRP-1 and VEGF mRNA expression in NCI-N87 and ST-2 cells

To determine the effects of EGF on NRP-1 and VEGF mRNA expression, NCI-N87 and ST-2 cells were grown to subconfluence in standard medium as described above, and the medium was changed to 5% FBS-containing medium overnight. Cells were then incubated with EGF (50 ng ml^−1^) for 4 or 24 h in 1% FBS-containing medium. Total RNA was extracted, and VEGF expression and NRP-1 expression were determined by Northern blot analysis.

### Determination of C225's effects on NRP-1 and VEGF mRNA induction by EGF in NCI-N87 cells

We evaluated the ability of C225 to block VEGF and NRP-1 mRNA induction by EGF. NCI-N87 cells grown under the conditions described above were pretreated with or without C225 (10 or 50 *μ*g ml^−1^) in 1% FBS-containing medium for 24 h, and EGF (50 ng ml^−1^) was then added. Cells were harvested after 4 or 24 h, and the relative expression levels of VEGF and NRP-1 mRNA were determined by Northern blot analysis.

### Determination of EGF's effects on Erk, Akt, and P38 phosphorylation in NCI-N87 cells

To determine the effect of EGF on the protein levels and phosphorylation of the signalling intermediates Erk, Akt, and P38 MAPK cells grown under the conditions described above were incubated with EGF (50 ng ml^−1^) for 0, 5, 10, 15, 30, or 60 min in 1% FBS-containing medium, and cell lysates were obtained. Phosphorylated and total protein levels were determined by Western blot analyses as described below.

### Determination of effects of Erk1/2, Akt, and P38 MAPK inhibition on VEGF and NRP-1 induction by EGF

To determine the effects of Erk1/2, Akt, and P38 MAPK inhibition on VEGF and NRP-1 induction, NCI-N87 cells grown under the conditions described above were pretreated with 50 *μ*M PD98059, 10 *μ*M U0126, 200 nM wortmannin, or 25 *μ*M SB203580 for 1 h in 1% FBS-containing medium, and then EGF (50 ng ml^−1^) was added for 24 h. Total RNA was extracted, and Northern blot analysis was performed. Preliminary experiments had shown that inhibitors at these concentrations blocked activation of their targets without inducing cell death (data not shown).

### RNA extraction and Northern blot analysis

Total RNA was extracted from cells using Tri Reagent following the manufacturer's instructions. Northern blot analysis was performed as previously described (Jung *et al*, 1999). A human VEGF-specific 204-bp cDNA probe was a gift from Dr Brygida Berse (Harvard Medical School, Boston, MA, USA), a human NRP-1-specific 639-bp cDNA probe was a gift from Dr Michael Klagsbrun (Harvard Medical School), and a glyceraldehyde-phosphate dehydrogenase (GAPDH) cDNA probe was purchased from American Type Culture Collection. The VEGF-specific probe identifies all alternatively spliced forms of its mRNA transcripts. Probes were purified by agarose gel electrophoresis using a QIAEX gel extraction kit (QIAGEN, Inc., Chatworth, CA, USA). Each cDNA probe was radiolabelled with [*α*-^32^P] deoxyribonucleotide triphosphate according to the random-primer technique using the Rediprime labelling system (Amersham Corp., Arlington Heights, IL, USA). Aliquots (25 g) of total RNA were subjected to electrophoresis in 1% denaturing formaldehyde–agarose gels. The RNA was transferred to a Hybond-N+ positively charged nylon membrane (Amersham Corp.) overnight by capillary elution and subjected to ultraviolet crosslinking at 120 000 *μ*J cm^−2^ using an ultraviolet Stratalinker 1800 (Stratagene, La Jolla, CA, USA). After blots were incubated for 3–4 h at 65°C in rapid hybridisation buffer (Amersham), the membranes were hybridised overnight at 65°C with the cDNA probe for VEGF, NRP-1, or GAPDH. The probed nylon membranes were washed and exposed to radiographic film (Life Technologies).

### Western blot hybridisation

For Western blot hybridisation, cells were rinsed twice with ice-cold phosphate-buffered saline and then lysed with protein lysis buffer (20 mM sodium phosphate (pH 7.4), 150 mM sodium chloride, 1% Triton X-100, 5 mM EDTA, 5 mM phenylmethylsulphonyl fluoride, 1% aprotinin, 1 *μ*g ml^−1^ leupeptin, and 500 *μ*M Na^3^VO^4^). The protein was quantitated spectrophotometrically using a BCA assay (Pierce, Rockford, IL, USA). Aliquots (50 *μ*g) of the protein were subjected to electrophoresis on 8–10% polyacrylamide gels. The protein was then transferred onto a nitrocellulose membrane (Schleicher and Schuell, Keene, NH, USA) by electrotransfer. Following blocking with 5% milk in 0.5% Tween 20 in phosphate-buffered saline, the membrane was probed with the primary antibody (1:1000 dilution of rabbit polyclonal anti-EGF-R antibody (Upstate Biotechnology, Waltham, MA, USA), mouse monoclonal antiphospho-specific p44/42 MAPK (Erk1/2) antibody, rabbit polyclonal antiphospho-specific Akt (site S473) antibody, or rabbit polyclonal antiphospho-specific P38 MAPK antibody (all from Cell Signalling Technology, Beverly, MA, USA)). The membranes were then washed and treated with the secondary antibody labelled with horseradish peroxidase (goat anti-rabbit or anti-mouse immunoglobulin (Amersham) at a 1:3000 dilution). Protein bands were visualised using a commercially available chemiluminescence kit (Amersham). For assaying total protein levels, the membrane was washed with stripping solution (100 mM 2-mercaptoethanol, 2% sodium dodecyl sulphate, and 62.5 mM Tris-HCl (pH 6.7)) for 30 min at 65°C and reprobed with rabbit polyclonal anti-p44/42 (Erk1/2), anti-Akt, or anti-p38 antibody (all at a 1:1000 dilution).

### Densitometric quantification

Densitometric analysis was performed using Image Quant software (Molecular Dynamics, Sunnyvale, CA, USA) to quantify the results of Northern blot analysis (VEGF, NRP-1, and GAPDH mRNA expression) in the linear range of the film. GAPDH mRNA was used as an internal control for loading Northern blots.

### Immunohistochemical analysis of human gastric cancer specimens

Expression of EGF-R and NRP-1 in human gastric cancer specimen was analysed immunohistochemically as previously described (Jung *et al*, 2002) with some modifications. For antigen retrieval, slides for EGF-R were treated with pepsin and incubated at 37°C for 20 min, slides for NRP-1 were placed in 0.1 M citrate buffer and heated in a microwave oven for 5 min. To intensify the signals, we used the Tyramide amplification method using the TSA kit (Perkin Elmer). Ten paraffin-embedded human gastric cancer specimens were stained for EGF-R and NRP-1 and the expression pattern was analysed at × 100 magnification for determination of colocalisation of proteins in serial sections.

## RESULTS

### Expression of NRP-1 and EGF-R by human gastric cancer cells

**Figure 1:**
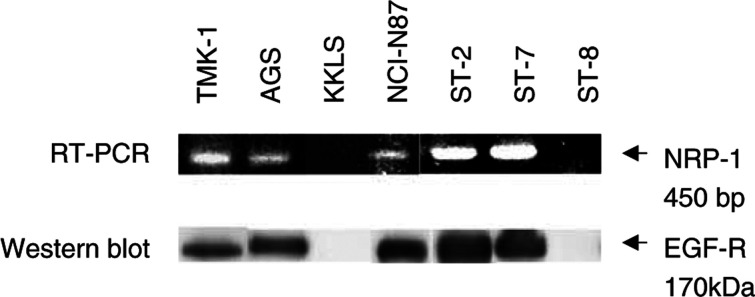
Expression of NRP-1 and EGF-R by seven human gastric cancer cell lines. Cells were grown to 80% confluence in 10% serum-containing medium. RT–PCR with NRP-1-specific primers was performed to detect NRP-1 RNA expression. Western blots were performed to detect EGF-R protein expression. NRP-1 expression closely mirrored EGF-R expression.

The expression of NRP-1 mRNA and EGF-R protein by human gastric cancer cell lines was examined by RT–PCR and Western blot analysis, respectively. RT–PCR revealed that five (TMK-1, AGS, NCI-N87, ST-2, ST-7) of seven gastric cancer cell lines constitutively expressed NRP-1 mRNA. NRP-1 expression was not detected in KKLS and ST-8 cells (Figure 1

). Northern blot hybridisation produced similar results (data not shown). The five NRP-1-positive cell lines also expressed EGF-R protein to various degrees, but EGF-R protein was not detected in the two NRP-1-negative cell lines (Figure 1).

### Effect of EGF on NRP-1 and VEGF expression in NCI-N87 and ST-2 cells

**Figure 2:**
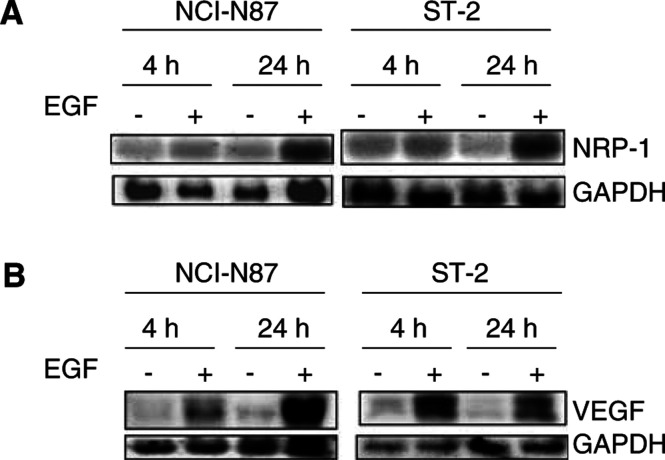
Induction of NRP-1 (**A**) and VEGF (**B**) in NCI-N87 and ST-2 cells after treatment with EGF. The cells were incubated in 5% serum-containing medium overnight and then were incubated with or without 50 ng ml^−1^ EGF for 4 or 24 h in 1% serum-containing medium. Total RNA was extracted for Northern blot analysis. EGF induced NRP-1 and VEGF mRNA expression at 4 and 24 h.

To analyse the regulation of NRP-1 expression by EGF, we studied NCI-N87 and ST-2 human gastric cancer cells, which expressed relatively high levels of EGF-R. Furthermore, others have shown that high levels of EGF-R protein are present in the membrane of NCI-N87 cells (Basque *et al*, 2001). ST-2 cells expressed relatively high levels of EGF-R protein (Figure 1) when treated with EGF for 4 or 24 h. Control cells were harvested at each time point to exclude any effects on gene expression from increasing cell confluence (Koura, 1996). NRP-1 mRNA expression was slightly induced at 4 h and strongly induced at 24 h by incubation with EGF (3.0-fold over control for NCI-N87; 3.3-fold over control for ST-2) (Figure 2A

). Similar NRP-1 induction by EGF was also observed in AGS cells (data not shown).

In preliminary experiments, we found that all gastric cell lines studied expressed VEGF mRNA at various levels (data not shown). We therefore examined the effect of EGF on VEGF mRNA induction using NCI-N87 and ST-2 cells. EGF induced VEGF mRNA expression at 4 h (2.3-fold over control for NCI-N87 cells; 3.8-fold over control for ST-2 cells), and the induction continued at 24 h (Figure 2B).

### Effect of C225 on NRP-1 and VEGF induction by EGF in NCI-N87 cells

**Figure 3:**
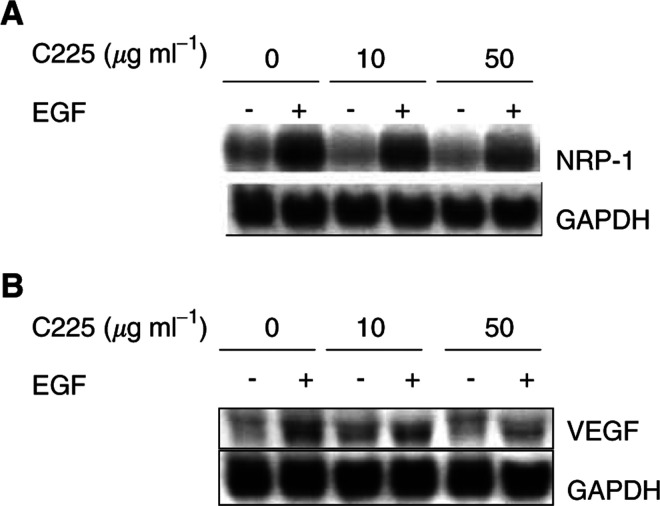
Inhibitory effect of C225 on NRP-1 (**A**) and VEGF (**B**) mRNA induction by EGF in NCI-N87 cells. The cells were incubated in 5% serum-containing medium overnight and then were pretreated with or without C225 (10 or 50 *μ*g ml^−1^) in 1% FBS-containing medium for 24 h. EGF (50 ng ml^−1^) then was or was not added, and cells were harvested after 4 or 24 h. Relative expression levels of VEGF and NRP-1 mRNA were determined by Northern blot analysis. Pretreatment of the cells with C225 inhibited EGF's effect in a dose-dependent manner.

We investigated the effect of blockade of EGF-R with C225 on the induction of NRP-1 and VEGF following treatment with EGF. Pretreatment of the cells with C225 (10 or 50 *μ*g ml^−1^) for 24 h followed by treatment with EGF inhibited induction of NRP-1 and VEGF in a dose-dependent manner (Figure 3

). At a C225 dose of 50 *μ*g ml^−1^, VEGF expression induced by EGF was nearly completely blocked (Figure 3B), whereas NRP-1 expression was only partially blocked (Figure 3A).

### Effect of EGF on signalling pathways involved in NRP-1 and VEGF induction in NCI-N87 cells

**Figure 4:**
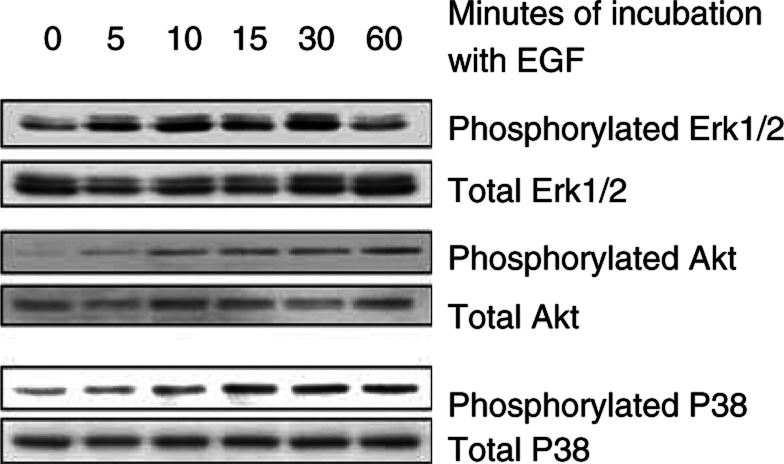
Effect of EGF on Erk1/2, Akt, and P38 phosphorylation in NCI-N87 cells. The cells were incubated in 5% serum-containing medium overnight and then were incubated with 50 ng ml^−1^ EGF for the indicated duration in 1% serum-containing medium. Phosphorylated and total protein levels were determined by Western blot analyses. EGF led to induction of phosphorylated Erk1/2, Akt, and P38.

To determine the signalling pathways induced by EGF in NCI-N87 cells, Western blot analysis was performed after incubation of cells with EGF for various durations. As shown in Figure 4

, a moderate increase in the phosphorylation of Erk1/2 began within 5 min of EGF treatment and returned to the basal level at 60 min. EGF also moderately increased Akt phosphorylation as early as 5 min of incubation, and the effect continued for at least 60 min. EGF only minimally increased phosphorylation of P38 MAPK 15 min after treatment. EGF also minimally increased phosphorylation of c-jun amino-terminal kinase (JNK) (data not shown). The relative expression levels of total Erk1/2, Akt, and P38 were not significantly altered after EGF treatment (Figure 4).

**Figure 5:**
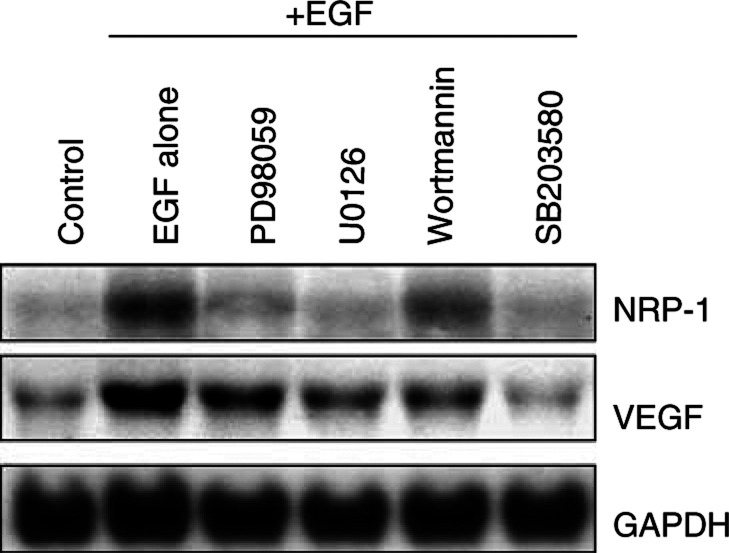
Effect of Erk1/2, Akt, and P38 MAPK inhibition on NRP-1 and VEGF induction by EGF in NCI-N87 cells. The cells were incubated in 5% serum-containing medium overnight and then were pretreated with 50 *μ*M PD98059, 10 *μ*M U0126, 200 nM wortmannin, or 25 *μ*M SB203580 for 1 h in 1% FBS-containing medium. EGF (50 ng ml^−1^) was then added for 24 h. Control cells were not treated with EGF (lane 1) and cells treated with EGF without addition of signalling inhibitors served as another internal control (lane 2). Total RNA was extracted, and Northern blot analysis was performed. Blockade of the Erk1/2, Akt or P38 pathways all led variable decreases in NRP-1 and VEGF mRNA expression.

We next selectively blocked the Erk1/2, Akt, or P38 MAPK pathways to determine which pathway was essential for EGF induction of NRP-1 and VEGF mRNA expression in NCI-N87 cells. The results of Northern blot analysis are shown in Figure 5

. PD98059, U0126, and SB203580 effectively inhibited NRP-1 induction by EGF, whereas wortmannin did not. In addition, SB203580 nearly completely abrogated VEGF induction by EGF, and wortmannin and U0126 partially blocked VEGF induction, to a similar degree. Thus, blockade of the Erk1/2 or P38 MAPK pathway effectively suppressed NRP-1 mRNA induction by EGF. VEGF mRNA induction was completely inhibited by blockade of the P38 pathway.

### Immunohistochemical analysis of human gastric cancer specimens for NRP-1 and EGF-R

**Figure 6:**
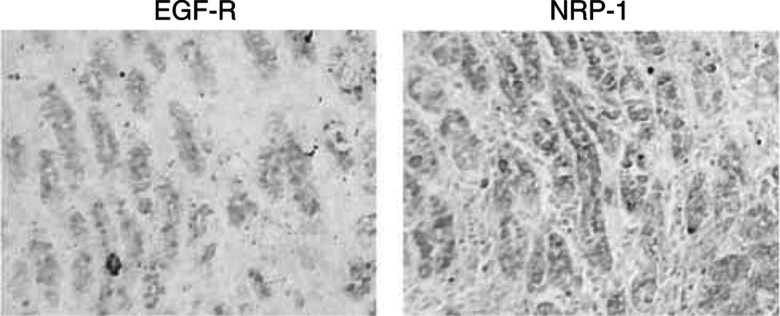
Immunohistochemical staining of intestinal-type human gastric cancer for EGF-R and NRP-1. EGF-R and NRP-1 were colocalised in a glandular pattern in a moderately differentiated gastric cancer specimen. Original magnification × 100.

We stained 10 paraffin-embedded human gastric cancer specimen for EGF-R and NRP-1 (Figure 6

). Six samples were differentiated (intestinal-type) and four were poorly differentiated (diffuse-type). Overall, eight of 10 specimens expressed nrp-1 in the tumour epithelial cells and nine of 10 expressed EGF-R. Among the differentiated tumours, half of them expressed nrp-1 and two-thirds expressed egf-r. Two of the six intestinal-type tumours demonstrated colocalisation of NRP-1 and EGF-R. Among the poorly differentiated tumours, half of them demonstrated colocalisation of NRP-1 and EGF-R.

## DISCUSSION

In the present study, we examined the expression of NRP-1 and EGF-R and the effect of EGF on NRP-1 and VEGF expression in human gastric cancer cells. Five of seven gastric cancer cell lines constitutively expressed NRP-1 mRNA. The expression pattern of NRP-1 was directly related to EGF-R protein expression, and EGF increased not only NRP-1 but also VEGF mRNA expression in two different cell lines. We also confirmed the expression pattern of NRP-1 and EGF-R in human gastric cancer specimens and found one-third of the differentiated cancers and half of the undifferentiated cancers demonstrated colocalisation of these two proteins (this implies an association, but causality cannot be confirmed by immunohistochemical studies of human specimens). These data suggest that regulation of NRP-1 expression is associated with EGF-R activation and that ligands for EGF-R could theoretically contribute to tumour angiogenesis by a mechanism that involves upregulation of VEGF and NRP-1 expression in this tumour system. Furthermore, EGF induction of NRP-1 and VEGF might involve multiple signalling pathways, thus providing multiple potential targets for inhibiting the induction of NRP-1 and VEGF. Further study is needed to validate the importance of EGF-R activation and NRP-1 induction.

NRP-1 is expressed on ECs and tumour cells. Various tumour cell types, such as breast and prostate cancers, express abundant levels of NRP-1 mRNA, whereas low levels of NRP-1 are found in some normal adult tissues (Soker *et al*, 1998). Moreover, NRP-1 expression appears to correlate with advanced stage or grade in prostate cancer and astrocytoma (Ding *et al*, 2000; Latil *et al*, 2000). In our study, five of seven gastric cancer cell lines expressed NRP-1 mRNA. The exact role of NRP-1 in tumour cells remains to be elucidated; however, it is possible that NRP-1 may augment tumour angiogenesis and/or tumour cell survival (Miao *et al*, 2000; Bachelder *et al*, 2001).

Little is known about the role of NRP-1 in tumour cells. NRP-1 is a nontyrosine kinase transmembrane protein (Soker *et al*, 1998). Unlike ECs, tumour cells may express NRP-1 as the only VEGF receptor. Overexpression of NRP-1 in prostate carcinoma cells enhances angiogenesis and increases proliferation of ECs, suggesting that the expression of NRP-1 by tumour cells themselves can influence tumour growth and angiogenesis (Miao *et al*, 2000). Furthermore, expression of NRP-1 in tumour cells enhances binding of VEGF-165 to these cells (Miao *et al*, 2000). It is possible that NRP-1 on tumour cells binds to VEGF-165 and acts as a carrier of VEGF to enhance VEGF binding to VEGF tyrosine kinase receptors on ECs. Alternatively, VEGF-165 might stimulate tumour cells directly via NRP-1 through an unknown mechanism. A recent study has shown that VEGF-165 acts as an autocrine survival factor in NRP-1-positive breast carcinoma cells lacking VEGFR-2 and that this likely occurs through activation of the PI-3 kinase pathway (Bachelder *et al*, 2001). It has also been reported that VEGF-165 may act as an autocrine growth factor in a VEGFR-positive human gastric cancer cell line (Tian *et al*, 2001). NRP-1 could act as a coreceptor that enhances VEGF-165 function in both VEGFR-2- and NRP-1-positive tumour cells. We investigated the effect of VEGF-165 on proliferation of tumour cells in NRP-1-positive NCI-N87, ST-2, and TMK-1 cells, but VEGF-165 had no effect on cell growth in these cells (data not shown).

VEGF expression is known to be regulated by numerous cytokines and growth factors (Akagi *et al*, 1998). In contrast, factors that regulate NRP-1 expression in ECs and tumour cells are not fully elucidated. Recent studies have shown that tumour necrosis factor-*α* and VEGF increase NRP-1 expression in human and bovine retinal ECs, respectively (Giraudo *et al*, 1998; Oh *et al*, 2002). Other investigators have shown that EGF increases NRP-1 expression in human malignant astrocytoma cell lines and that the expression of NRP-1 mRNA peaks at 4 h and returns to basal levels 8 h after EGF treatment (Ding *et al*, 2000). In our study, NRP-1 mRNA expression was induced by EGF treatment at 24 h in three different gastric cancer cell lines (AGS, NCI-N87, and ST-2). Further experiments revealed that EGF also increased VEGF mRNA expression in NCI-N87 and ST-2 cells but not in AGS cells (data not shown for AGS cells). The factors involved in the regulation of VEGF may thus be dependent upon the tumour system under study (Akagi *et al*, 1998). In TMK-1 cells (cells that express relatively low levels of EGF-R protein), EGF did not increase either NRP-1 or VEGF (data not shown).

C225, an anti-EGF-R monoclonal antibody, binds EGF-R with affinity similar to that of EGF and is able to block EGF binding and EGF activation of the receptor. EGF-R signalling is critical for cell proliferation. Furthermore, EGF-R-mediated signals contribute to other processes that are crucial to cancer progression, including angiogenesis, metastatic spread, and the inhibition of apoptosis (Kim *et al*, 2001). Recently, several studies have demonstrated that *in vitro* treatment of cancer cells with C225 can downregulate the production of angiogenic factors such as VEGF, interleukin-8, or basic fibroblast growth factor and that *in vivo* inhibition of EGF-R results in growth inhibition and reduction in microvessel density accompanied by decreases in angiogenic factor expression (Petit *et al*, 1997; Perrotte *et al*, 1999; Bruns *et al*, 2000; Ciardiello *et al*, 2000). These results strongly support the involvement of the EGF-R signalling pathways in the regulation of angiogenesis and suggest that C225 could have therapeutic utility, not only through its ability to inhibit the growth of tumour cells but also through its ability to suppress tumour angiogenesis. In our study, C225 blocked both VEGF and NRP-1 mRNA induction by EGF in a dose-dependent manner in NCI-N87 cells.

EGF has been shown to influence various signalling pathways, including ras–raf–MEK–MAPKs, PI-3 kinase/Akt, and the stress-activated protein kinases (SAPKs or JNKs) (Yarden and Sliwkowski, 2001). We demonstrated that, in NCI-N87 cells, the Erk1/2, Akt, and P38 MAPK pathways are activated by EGF treatment. The modest level of activation may be secondary to the relative moderate levels of EGF-R compared to other cell types (Figure 1). Blockade of the Erk1/2 or P38 MAPK pathway inhibited EGF-induced NRP-1 expression more effectively than did inhibition of the Akt pathway. In addition, blockade of P38 activity nearly completely inhibited EGF-induced VEGF expression, and blockade of the Erk1/2 or Akt pathway partially inhibited VEGF induction. Taken together, the data suggest that the signalling mechanisms leading to NRP-1 induction by EGF are distinct from those that induce VEGF. However, blockade of P38 MAPK inhibited EGF induction of both NRP-1 and VEGF and might be a therapeutic target in this tumour system. The P38 pathway has not been extensively investigated in angiogenic systems; however, we previously found that treatment of human vascular smooth muscle cells with interleukin-1*β* leads to VEGF induction via P38 MAPK activation (Jung *et al*, 2001). Others have also shown that P38 can be phosphorylated by EGF-R activation (Kanda *et al*, 2001; Yamanaka *et al*, 2001). Taken together, our studies along with others support the role of EGF-R activation of angiogenic pathways through P38. Thus, P38 may be a common angiogenic signalling pathway in multiple cell types.

The mechanisms by which the EGF-R signalling pathways regulate VEGF and NRP-1 are unclear. Stimulation of the EGF-R signalling pathways is known to activate ras and raf, resulting in phosphorylation of c-fos and c-jun and leading to increased AP-1 transcriptional activity. The VEGF promoter has several AP-1 binding sites and increased AP-1 activity leads to transcription of VEGF (Rozakis-Adcock, 1993; Kerbel *et al*, 1998). The PI-3 kinase pathway also plays a role in VEGF induction by EGF-R signalling (Maity *et al*, 2000).

Studies in an astrocytoma cell line showed that activation of p21-Ras induces not only VEGF but also NRP-1 expression (Ding *et al*, 2000). A recent study has shown that NRP-1 is the downstream target of transcription factor Ets-1 in ECs (Teruyama *et al*, 2001). VEGF is a potent inducer of Ets-1 in ECs, and this induction of Ets-1 is mediated by the activation of Erk1/2 (Tanaka *et al*, 1999).

In summary, we have shown that EGF and EGF-R play a role in the regulation of NRP-1 and VEGF expression via multiple signalling pathways in human gastric cancer cells. Further studies are required to determine the clinical importance of activation of the EGF-R signalling pathways and the downstream effect on VEGF and NRP-1 expression.
